# The N-Terminal Part of the Dishevelled DEP Domain Is Required for Wnt/β-Catenin Signaling in Mammalian Cells

**DOI:** 10.1128/MCB.00145-17

**Published:** 2017-08-28

**Authors:** Petra Paclíková, Ondřej Bernatík, Tomasz Witold Radaszkiewicz, Vítězslav Bryja

**Affiliations:** a Department of Experimental Biology, Faculty of Science, Masaryk University, Brno, Czech Republic; b Institute of Biophysics, The Czech Academy of Sciences, Brno, Czech Republic

**Keywords:** CRISPR/Cas, DEP domain, Dishevelled, Wnt/β-catenin signaling, Wnt3a

## Abstract

Dishevelled (DVL) proteins are key mediators of the Wnt/β-catenin signaling pathway. All DVL proteins contain three conserved domains: DIX, PDZ, and DEP. There is a consensus in the field that the DIX domain is critical for Wnt/β-catenin signaling, but contradictory evidence regarding the function of the DEP domain exists. It has been difficult, until recently, to test the importance of the DEP domain rigorously because of the interference with endogenous DVL, expressed in all Wnt-responsive cell lines. In this study, we took advantage of DVL knockout (DVL1/DVL2/DVL3 triple knockout) cells fully deficient in Wnt3a-induced signaling events and performed a series of rescue experiments. Using these complementation assays, we analyzed the role of individual DVL isoforms. Further domain mapping of DVL1 showed that both the DVL1 DEP domain and especially its N-terminal region are required and sufficient for Wnt3a-induced phosphorylation of LRP6 and TopFlash reporter activation. On the contrary, multiple DEP domain mutants deficient in the planar cell polarity (PCP) pathway could fully rescue the Wnt3a response. This study provides conclusive evidence that the DVL DEP domain is essential for Wnt/β-catenin signaling in mammalian cells and establishes an experimental system suitable for further functional testing of DVL.

## INTRODUCTION

The Wnt/β-catenin pathway (also referred to as the “canonical pathway”) is an evolutionarily conserved cellular signaling pathway that controls embryonal development and adult homeostasis. Deregulation of Wnt signaling is causative for the development of several types of cancer and other diseases (1).

Dishevelled (DVL1, DVL2, and DVL3 in mammals; Dsh in Drosophila) is a key component of the downstream relay mechanism of Wnt signaling pathways, and it is also one of the components shared between most branches of Wnt signaling. DVL, which acts as a scaffold protein, mediates integration of the signal that is received from the receptor complex(es) (2–4). Structurally, DVL is a modular protein that contains three well-defined domains linked by the intrinsically disordered regions. The N-terminal DIX (Dishevelled, axin) domain is connected with the central PDZ (postsynaptic density, discs large, zonula occludens) domain by the serine (Ser)/threonine (Thr)-rich unstructured region. The PDZ domain and the C-terminally localized DEP (DVL, Egl-10, pleckstrin) domain are linked by the region enriched in proline (Pro-rich region). These features enable DVL to modulate and transduce signals through the different channels of downstream Wnt signaling pathways.

In the Wnt/β-catenin pathway, DVL is essential for the phosphorylation of the intracellular domains of LRP5 and LRP6. Phosphorylated LRP5/6 can inhibit the function of the β-catenin destruction complex, composed of axin1, glycogen synthase kinase 3 (GSK3), and adenomatous polyposis coli (APC) (5, 6). There is conclusive evidence showing that DIX domain-mediated DVL polymers, formed in the head-to-tail manner (7), are a physical platform for the formation of signalosomes, complexes of DVL, and other signaling components that are required for LRP6 phosphorylation (8). The requirement for the DIX domain for the phosphorylation of LRP6 and its capacity to multimerize seem to be the molecular reason for the absolute requirement for the DIX domain in Wnt/β-catenin signaling.

The analysis of the DVL regions required for the Wnt/β-catenin pathway by rescue and gain-of-function (GOF) experiments showed that the DEP domain is largely dispensable for activation of downstream signaling in the Wnt/β-catenin pathway (9, 10). On the other hand, conclusive work in Drosophila demonstrated that the DEP domain of Dsh is responsible for the other well-described function of Dsh, which is in the Wnt/planar cell polarity (PCP) pathway. The Wnt/PCP pathway is a molecular mechanism that controls asymmetric localization and the maintenance of polarity in the epithelial sheet (for a review, see reference 11). The Dsh DEP domain is required for the association of Dsh with the membrane, an event that underlies the asymmetric subcellular distribution of PCP proteins and a functional Wnt/PCP pathway (9, 12).

It should be noted, however, that the question about the requirement for the DVL DEP domain in the endogenous Wnt–Frizzled (FZD)–DVL–β-catenin axis in mammalian systems still has not been fully resolved because of two experimental obstacles. First, the GOF experiments have been performed in wild-type (WT) cells, where endogenous DVL, multimerizing with the overexpressed variants, could provide the missing DEP domain (if required). Second, overexpressed DVL can bypass the requirement for the Wnt/receptor interaction and other proximal events by activating the pathway downstream of the receptor complex. In this study, we attempted to address this issue by complementation assays performed in HEK293 cells that lacked endogenous DVL and that were unable to respond to Wnt3a. Using this experimental system, we performed a set of rescue experiments that convincingly show that the DEP domain and especially its N-terminal part are required for Wnt3a-induced activation of Wnt/β-catenin signaling in mammalian cells.

## RESULTS

**FIG 1:**
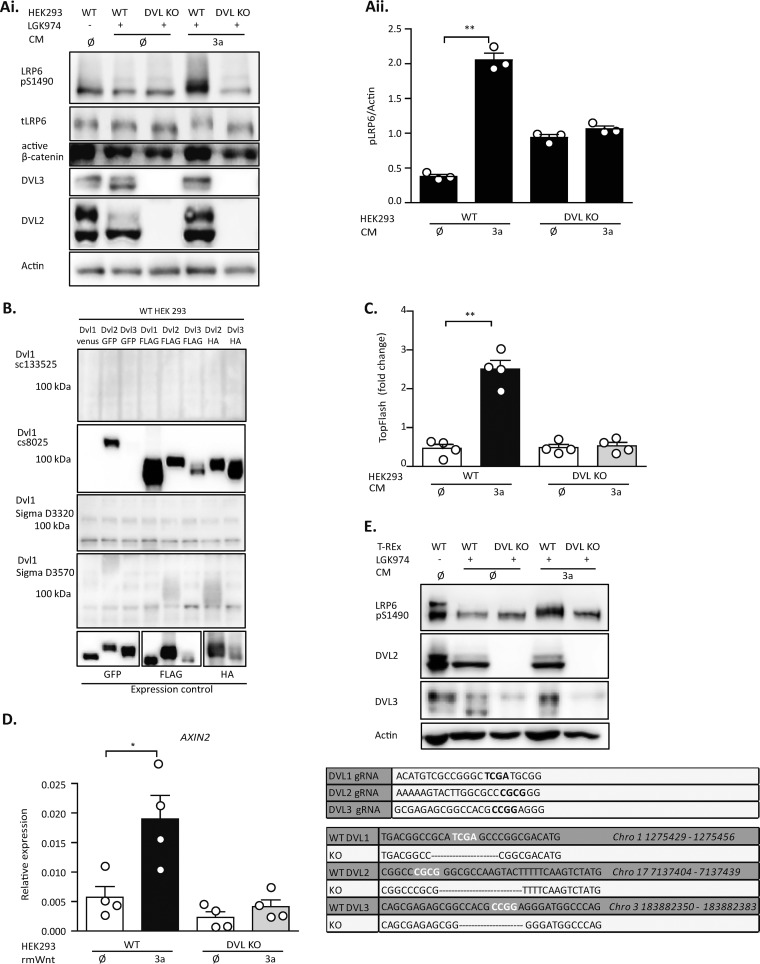
DVL-deficient cells are unable to respond to Wnt3a. Both wild-type (WT) and *DVL1/DVL2/DVL3* triple knockout (DVL KO) HEK293 cells were treated with Porcupine inhibitor (LGK974) for 24 h to reduce autocrine signaling, and then the cells were treated for 2 h (for Western blotting [WB]) or for 14 h (for the TopFlash reporter assay) by control (ø) or Wnt3a (3a) conditioned medium (CM). (A) In DVL KO HEK293 cells, S1490-LRP6 phosphorylation and active β-catenin were not induced by Wnt3a; total LRP6 (tLRP6 ) levels were not changed. A lack of DVL2 and DVL3 in DVL KO HEK293 cells served as a control for cell identity. (i) Western blots; (ii) quantification of density of pS1490-LRP6 compared to that of actin (*n* = 3). (B) DVL1 antibody testing. DVL KO HEK293 cells were transfected according to the loading scheme and analyzed by WB. None of the anti-DVL1 antibodies was able to detect DVL1 specifically. All DVL constructs were expressed to a comparable level, as demonstrated by antitag (GFP, FLAG, and HA) staining. (C) TCF/LEF-dependent transcription is not induced in DVL KO HEK293 cells, as analyzed by the TopFlash reporter assay (*n* = 4). (D) DVL KO HEK293 cells are not able to induce the expression of *AXIN2* after Wnt3a treatment, as quantified by RT-PCR (*n* = 4). Cells were treated with mouse Wnt3a recombinant protein (rmWnt; 100 ng/ml). (E) (Top) Analysis of cells of the DVL KO HEK293 T-REx cell line. DVL KO T-REx cells are not able to phosphorylate S1490-LRP6 after Wnt3a induction. A lack of DVL2 and DVL3 in DVL KO HEK293 cells served as a control for cell identity. (Bottom) The guide RNA (gRNA) design and the sequences of verified deletions in the DVL KO T-REx cell line edited by the CRISPR/Cas9 system are shown. Analysis for statistically significant differences was performed by paired Student's *t* test (*, *P* < 0.05; **, *P* < 0.01).

As the first step, we analyzed the ability of wild-type (WT) and *DVL1/DVL2/DVL3* triple knockout (KO) HEK293 cells (DVL KO HEK293 cells; characterized by us and others earlier [13, 14]) to respond to Wnt3a, a prototypical ligand of the Wnt/β-catenin signaling pathway. In all experiments, HEK293 cells were pretreated with LGK974 (15), a Porcupine inhibitor that prevents the production of all Wnt ligands, including noncanonical Wnt5a. This LGK974 treatment was used to reset the system in order to eliminate the variability caused by autocrine production of Wnt5a ligands that interfere with the action of the Wnt3a pathway receptor. The experiment clearly demonstrated that DVL KO HEK293 cells are fully deficient in their ability to respond to Wnt3a, a phenomenon that we confirmed using several well-described pathway activation readouts. First, Wnt3a was unable to induce the phosphorylation of LRP6 on serine 1490 (pS1490-LRP6) (Fig. 1Ai; quantified in Fig. 1Aii) or to increase the amount of dephosphorylated β-catenin detected by anti-active β-catenin antibody in DVL KO HEK293 cells (Fig. 1Ai). The identity of the cell types used was confirmed by the absence of DVL2 and DVL3 in DVL KO HEK293 cells. Note that, to our knowledge, there is no commercially available antibody capable of specifically detecting the endogenous levels of DVL1 in HEK293 cells (for the validation of antibody specificity, see Fig. 1B). In agreement with the results of the biochemical analysis, Wnt3a was unable to activate TCF/LEF-driven transcription in DVL KO HEK293 cells, as analyzed by the TopFlash reporter assay (Fig. 1C) and quantitative reverse transcription-PCR (RT-PCR) of the Wnt target gene, *AXIN2* (Fig. 1D). In order to exclude possible off-target effects, we repeated the experiments in a separately established DVL KO cell line generated by clustered regularly interspaced short palindromic repeat (CRISPR)/Cas9 editing in HEK293 T-REx cells. Successful targeting of the *DVL1*, *DVL2*, and *DVL3* loci was confirmed by sequencing (Fig. 1E, bottom). In agreement with the observations in HEK293 cells, even T-REx *DVL1/DVL2/DVL3* triple-null cells (DVL KO T-REx cells) were unable to respond to Wnt3a, as analyzed by the pS1490-LRP6 shift assay (Fig. 1E). These cells were later used as an independent control to confirm the key findings presented further.

**FIG 2:**
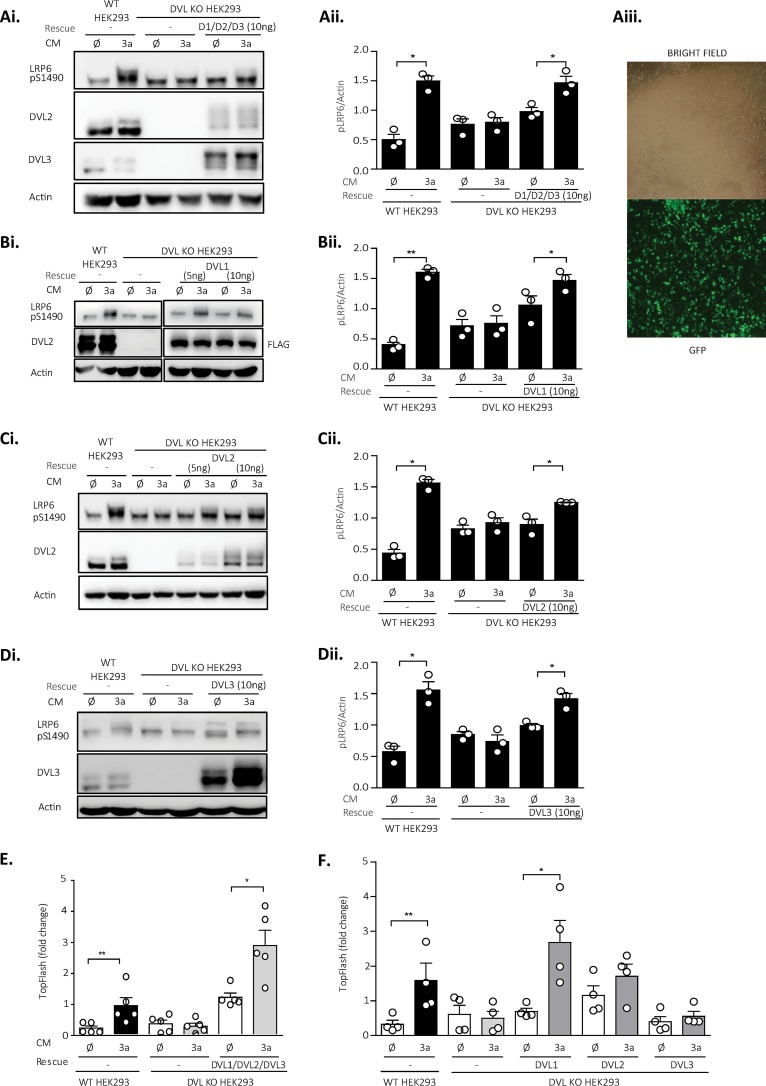
DVL overexpression rescues the Wnt3a response in DVL KO HEK293 cells. (A to F) Cells were transfected with the indicated combinations of plasmids (the concentration of each plasmid carrying DVL was 10 ng/sample, if not stated otherwise) and on the next day were treated with control (ø) or Wnt3a (3a) conditioned medium (CM) for 2 h (for Western blotting [WB]) or 14 h (for the TopFlash reporter assay). The response to Wnt3a was monitored by WB (S1490-LRP6) (Ai to Di; *n* = 3) and the TopFlash reporter system (*n* = 5 for panel E; *n* = 4 for panel F). pS1490-LRP6 signals were quantified by densitometry and normalized to those for actin (Aii to Dii; *n* = 3). (Aiii) Transfection efficiency was monitored by cotransfection of GFP. Analysis for statistically significant differences was performed by paired Student's *t* test (*, *P* < 0.05; **, *P* < 0.01).

The phenotypes of the DVL KO HEK293 cells described in Fig. 1 provided us with readouts for the subsequent rescue experiments, where we used Wnt3a-induced S1490-LRP6 phosphorylation (pS1490-LRP6) and the TopFlash reporter assay (16) as primary readouts of efficient signalosome formation (pS1490-LRP6) and downstream signaling (TopFlash reporter assay). The rescue experiments were performed by transient transfection, which was optimized to fulfill two basic criteria: (i) DVL expression levels had to be comparable to the endogenous expression levels in WT HEK293 cells, and (ii) the transfection efficiency had to be very high so that the majority (>80%) of cells were transfected, as estimated by the green fluorescent protein (GFP) signal (Fig. 2Aiii). GFP cotransfection was used as a control for transfection under the experimental conditions, and only when the criteria specified above were met, the samples were subjected to the subsequent analysis. Using these optimized conditions (see Materials and Methods), we were partially able to rescue the pS1490-LRP6 response to Wnt3a by simultaneous reintroduction of DVL1, DVL2, and DVL3 into DVL KO HEK293 cells (Fig. 2Ai; quantified in Fig. 2Aii).

As the next step, we tested the individual DVL isoforms separately for their capacity to rescue pS1490-LRP6 in DVL KO HEK293 cells after treatment with Wnt3a (Fig. 2B to D). This experiment revealed that all three DVL isoforms do rescue pS1490-LRP6, albeit to various extents, with DVL3 being the weakest rescuer. DVL3 was only partially able to rescue the effects of Wnt3a even at doses exceeding endogenous DVL3 levels (Fig. 2D). The expression level of DVL was monitored by the use of isoform-specific antibodies, except for DVL1, for which, to our knowledge, no suitable commercial antibody exists (Fig. 1B). To verify not only that our approach rescues pS1490-LRP6 but also that controlled DVL overexpression enables relay of the signal downstream, we analyzed the ability of DVL to rescue the Wnt3a-induced TCF/LEF-dependent transcription of DVL KO HEK293 cells using the TopFlash reporter assay. As shown in Fig. 2E, combined reexpression of all three DVL proteins restored this capacity, and the same could be recapitulated when DVL1 but not DVL2 and DVL3 were expressed individually (Fig. 2F).

**FIG 3:**
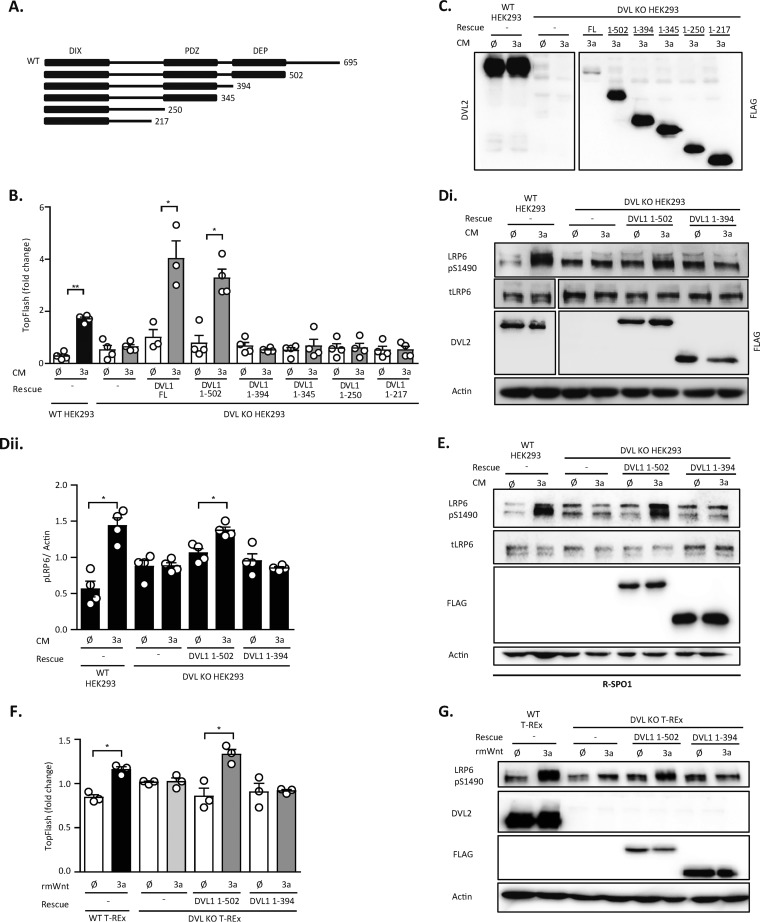
The DVL1 DEP domain is required for the Wnt3a response. (A) Schematic representation of DVL1 deletion mutants used for the rescue experiments; the DIX, PDZ, and DEP domains are indicated. (B to E) HEK293 cells were transfected with the indicated plasmids and treated according to the scheme; in the assay whose results are shown in panel E, cells were sensitized with R-spondin 1 (R-SPO1) to increase the response to Wnt3a. (B) Full-length (FL) DVL1 and the DVL1(1–502) mutant are able to rescue the transcriptional response in DVL KO HEK293 cells induced by Wnt3a conditioned medium (CM), as analyzed by the TopFlash reporter assay (*n* = 4). (C) Control of expression for the DVL1 mutants used in the assay whose results are shown in panel B was performed by Western blotting for the FLAG tag. (D, E) The DVL1(1–502) deletion mutant can restore the capacity of Wnt3a CM to induce the phosphorylation of pS1490-LRP6; tLRP6 (total LRP6) levels were not changed (Di, E). (Dii) Quantification by densitometry (*n* = 4). Actin was used as a loading control. (F, G) Rescue experiments in DVL KO T-REx cells. TCF/LEF-dependent transcription (F) and S1490-LRP6 phosphorylation (G) after transfection of the DVL1(1–502) mutant and the DVL1(1–394) mutant lacking the DEP domain were determined. The DVL1(1–394) mutant was not able to rescue either TCF/LEF-dependent transcription or S1490-LRP6 phosphorylation after Wnt3a treatment. Analysis for statistically significant differences was performed by paired Student's *t* test (*, *P* < 0.05; **, *P* < 0.01).

DVL1, which showed the best capacity to rescue the aforementioned phenotypes, was used in subsequent experiments to define the requirement for individual DVL domains/regions. We tested a series of DVL1 deletion mutants (schematized in Fig. 3A), which were described earlier (17). Interestingly, only full-length (FL) DVL1 and DVL1 from amino acids (aa) 1 to 502 [DVL1(1–502)], which lacked the C terminus, were sufficient to restore TopFlash reporter activation (Fig. 3B) and pS1490-LRP6 (Fig. 3Di; quantified in Fig. 3Dii) responses to Wnt3a. DVL1 mutants with further truncations, namely, the DVL1 mutant containing aa 1 to 394 [DVL1(1–394)], the DVL1 mutant containing aa 1 to 345, the DVL1 mutant containing aa 1 to 250, and the DVL1 mutant containing aa 1 to 217, were not able to do so, despite being expressed to comparable levels (Fig. 3C and D). In order to improve the signal-to-noise ratio, especially to enhance changes in pS1490-LRP6 and to better test the ability of individual DVL1 mutants to rescue pS1490-LRP6 or TCF/LEF-driven transcription, we pretreated cells with R-spondin 1 (R-SPO1). R-SPO1 inhibits internalization of receptor complexes, increases the surface levels of FZD, sensitizes cells to Wnt signaling, and, thus, enhances the response to Wnt3a (18). R-SPO1 addition improved the signal-to-noise ratio of S1490-LRP6 phosphorylation and confirmed that only the DVL1(1–502) mutant and not the DVL1(1–394) mutant could restore the phosphorylation of S1490-LRP6 after Wnt3a (Fig. 3E). We observed the same result in DVL KO T-REx cells, in which the response to Wnt3a was efficiently restored by DVL1(1–502) but not DVL1(1–394) both at the level of the TopFlash reporter activation and at the level of pS1490-LRP6 (Fig. 3F and G). Altogether, these data suggest that the DEP domain of DVL1 has a crucial function in the Wnt/β-catenin pathway and the cellular response to Wnt3a.

**FIG 4:**
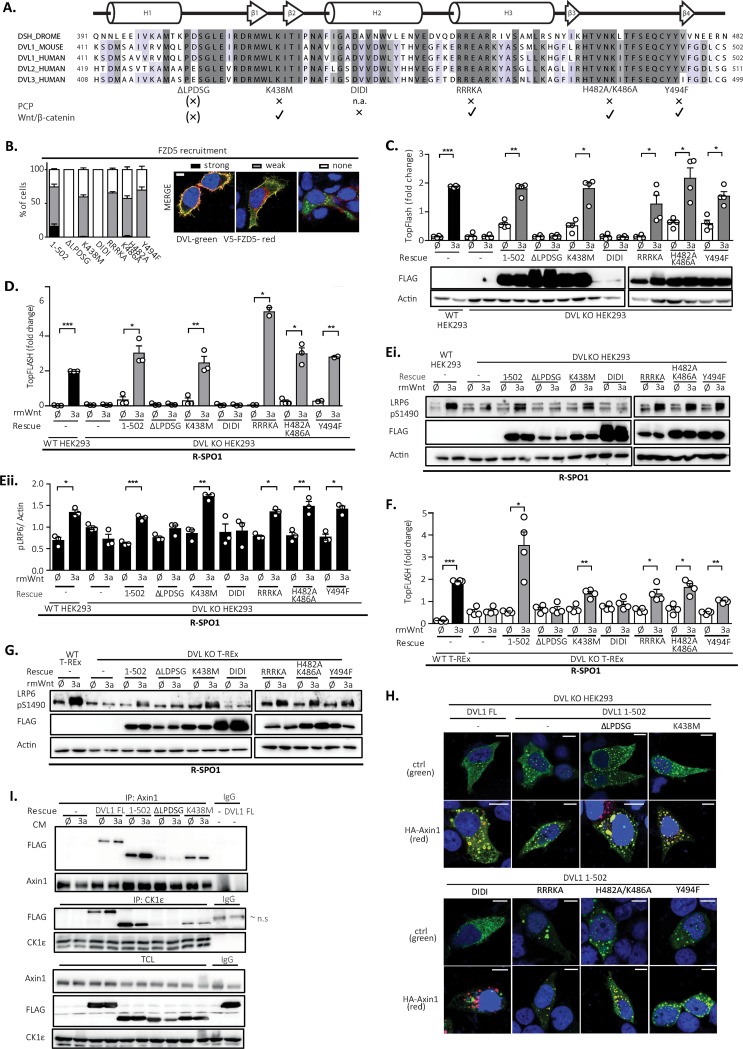
PCP pathway-specific DEP domain functional units are dispensable for the rescue of the cellular response to Wnt3a. (A) Schematic representation of the DVL1 DEP domain mutants used for the rescue experiments. The alignment of the sequences of individual human DVL isoforms with the Drosophila melanogaster (DROME) Dsh sequence and a summary of previously published Wnt/β-catenin or PCP pathways phenotypes of individual mutants (9, 10, 12, 19–21) are provided. All mutants were generated in the DVL1(1–502) mutant background. (B) (Left) Relocalization of individual DVL mutants to the membrane after cotransfection with Frizzled 5 (FZD5). (Right) Three categories of DVL1 localization after FZD5 coexpression were established: strong, weak, and no membrane localization of DVL1. One hundred cells were counted per condition (*n* = 3). All mutants showed reduced membrane recruitment compared to that for the control, the DVL1(1–502) mutant. Bar, 7.5 μm. (C to E) HEK293 cells were transfected as indicated and analyzed by the TopFlash reporter assay (C, D) (*n* = 4 for panel C, *n* = 3 for panel D) or WB (Ei, blots; Eii, quantification; *n* = 3). In the assay whose results are presented in panels D and E, cells were pretreated with R-spondin 1 (R-SPO1) and the DIDI mutant was transfected in a 100-ng dose so that its level equaled the protein levels of the other DVL1 mutants. All DVL1 DEP domain mutants except for the ΔLPDSG and DIDI mutants rescued the response to Wnt3a to an extent comparable to that for the DVL1(1–502) mutant. (F and G) Analysis of DEP mutants in DVL KO T-REx cells. All mutants except the ΔLPDSG and DIDI mutants were able to restore Wnt/β-catenin-dependent transcription, as analyzed by the TopFlash reporter assay (*n* = 4) (F) and S1490-LRP6 phosphorylation (G). Cells for which the results are shown in panels F and G were pretreated with R-spondin1 (R-SPO1). (H) The pattern of localization of full-length (FL) DVL1 and the individual DVL mutants resembles that of the DVL1(1–502) mutant. Colocalization with axin1 was determined by immunocytofluorescence. All mutants except DIDI colocalized with axin1. (Top) DVL1 only (green); (bottom) DVL1 (green) and axin1 (red). ctrl, control. Bars, 7.5 μM. (I) Coimmunoprecipitation of DVL1 mutants in the pulldown of endogenous axin1 and CK1ε. Cells were treated as indicated; unspecific IgG was used as a negative control. Analysis for statistically significant differences was performed by paired Student's *t* test (*, *P* < 0.05; **, *P* < 0.01; ***, *P* < 0.001).

The function of the DVL DEP domain in the Wnt/PCP pathway is well described, where several residues/surfaces in the DEP domain have been identified to be crucial for DVL function in the PCP pathway (9, 12, 19, 20). It is, however, not clear if and how these regions contribute to the newly identified function of the DEP domain for Wnt/β-catenin signaling. In order to address this issue, we mutated the following previously defined DEP domain functional units: (i) we mutated a positively charged region of the DVL1 DEP domain essential for binding to the negatively charged lipids in the plasma membrane required for PCP phenotypes in Drosophila mutant R464A/R465A/R468A/K469A (RRRKA) or H482A/K486A (19); (ii) the fully conserved Lys438 was mutated to Met (K438M), corresponding to the *Dsh*^*1*^ mutant (K417M) in Drosophila (9, 12); (iii) Tyr494 (Y473 in Drosophila), phosphorylated by the Abelson family kinasees (Abl) and essential for the PCP pathway in the fly, was mutated to phenylalanine (Y494F) (20); (iv) the N-terminal part of the DEP domain from aa 424 to 428 (LPDSG), proposed to be required and sufficient for the rescue of Wnt/β-catenin phenotypes in Drosophila (9) and secondary axis formation in Xenopus (10), was deleted (ΔLPDSG); and (v) the DEP domain region, proposed to be an interaction interface with Frizzled (21), was blocked in the D449I/D452I (DIDI) mutant. All mutant variants were made in the DVL1(1–502) mutant background, and their known functions are summarized in Fig. 4A. DEP domain mutants of DVL1 (Fig. 4A) are expected to be deficient in FZD-mediated membrane recruitment. Indeed, validation by the Frizzled 5 (FZD5)-induced translocation assay confirmed that all the mutants were defective, albeit to different extents, in their ability to get recruited to the membrane by FZD5 (Fig. 4B). Both the ΔLPDSG and DIDI mutants failed completely, whereas the PCP-specific mutants showed only a partial defect, as previously described (9, 19, 20).

In the next step, we tested the functional capacity of individual mutants in the complementation assay in the absence (Fig. 4C) and presence of R-SPO1 (Fig. 4D and E). Rescue experiments with the DEP domain mutants showed that only the ΔLPDSG and DIDI mutants failed to restore the Wnt3a response, as analyzed by the TopFlash reporter assay (Fig. 4C). All the mutants were expressed at comparable levels, with the exception of DIDI, which possibly did not fold well. Even when the levels of the DVL1 DIDI mutant were adjusted by transfection of larger amounts of DNA to match the levels of WT DVL1, the DVL1 DIDI mutant was still unable to rescue the Wnt3a-driven transcriptional response (data not shown). When DVL KO HEK293 cells were sensitized by R-SPO1, we could observe similar results both in the TopFlash reporter assay (Fig. 4D) and in the pS1490-LRP6 assay (Fig. 4Ei; quantified in Fig. 4Eii). Matching results were obtained using independent cell line DVL KO T-REx (Fig. 4F and G). In summary, these results convincingly confirmed that all the tested PCP mutants could restore the response to Wnt3a, but this was not the case for the ΔLPDSG and DIDI mutants, which likely interfere with the DEP domain function in a different way.

Interestingly, all the mutants, with the exception of the DIDI mutant, could efficiently colocalize with axin1 in the overexpression setup (Fig. 4H). This suggests that the ability to mediate the Wnt3a response and the ability to colocalize with axin1 do not always correlate. To get a better insight into this connection, we tested how individual DEP domain mutants interact with endogenous Wnt signaling components axin1 and CK1ε in response to Wnt3a by coimmunoprecipitation (co-IP) (Fig. 4I). We performed semiendogenous immunoprecipitation of FL DVL1 and the DVL1(1–502), ΔLPDSG, and K438M mutants with either axin1 or CK1ε. All these DVL1 variants interacted with the endogenous axin1 or CK1ε, but the ΔLPDSG mutant showed much weaker binding than the others. Wnt3a stimulation further enhanced the co-IP of axin1 and FL DVL1 and the DVL1(1–502) variant but not that of the ΔLPDSG mutant. This suggests that the inability to restore the response to Wnt3a correlates with the reduced capacity to interact in a dynamic manner with axin1.

**FIG 5:**
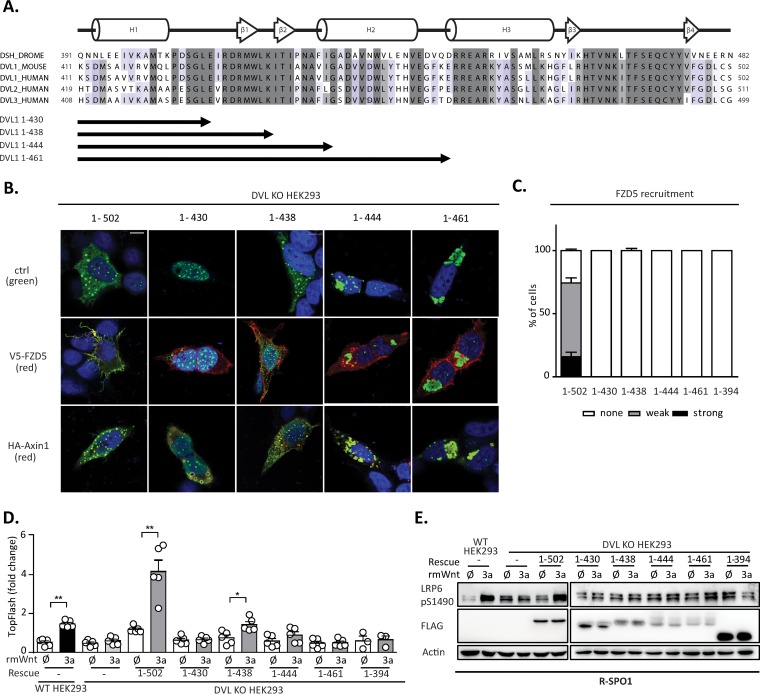
(A) Schematic representation of the DVL1 DEP domain truncation mutants used for the rescue experiments. (B) DVL KO HEK293 cells were transfected with the indicated DVL1 mutants and V5-FZD5 or HA-axin1 and stained by immunocytochemistry. (Top) DVL1 only; (middle) DVL1 (green) and FZD5 (red); (bottom) DVL1 (green) and axin1 (red). Bar, 7.5 μm. (C) Quantification of DVL1 localization after FZD5 cotransfection. The evaluation criteria used were the same as those described in the legend to Fig. 4B. None of the truncation mutants was recruited to the membrane by FZD5. (D) TopFlash reporter assay analysis of DVL1 mutants with the DEP domain truncation (*n* = 4). Only the DVL1(1–438) mutant showed a weak ability to rescue TCF/LEF-dependent transcription. (E) WB analysis of expression levels of individual mutants and their ability to rescue phosphorylation of S1490-LRP6 (*n* = 3). None of the mutants could rescue S1490-LRP6 phosphorylation. Analysis for statistically significant differences was performed by paired Student's *t* test (*, *P* < 0.05; **, *P* < 0.01).

The results of the analysis presented in Fig. 4 suggest that the N-terminal part of the DEP domain containing the LPDSG motif is required to restore the response to Wnt3a in DVL KO HEK293 cells. This implies that this region contains a sequence or structural motif necessary for the response to Wnt3a. But is such motif also sufficient? And what is the minimal region of the DEP domain that is sufficient to mediate the response to Wnt3a? In order to address these questions, we generated a new series of truncation mutants based on the structure of the DVL DEP domain: DVL1(1–430), whose sequence ends directly after the LPDSG amino acid sequence; DVL1(1–438), whose sequence ends after amino acid K438; DVL1(1–444), whose sequence ends before helix 2; and DVL1(1–461), whose sequence ends after helix 2 of the DEP domain (Fig. 5A). When DVL1(1–430) was overexpressed (Fig. 5B), it showed a strict nuclear localization, whereas the DVL1(1–444) and DVL1(1–461) mutants formed large aggregates, suggesting that these two variants did not fold properly. Only the DVL1(1–438) mutant showed a localization pattern similar to that of DVL1(1–502), which was used as a control. The DVL1(1–438) mutant also efficiently colocalized with axin1 (Fig. 5B), but similarly to the other truncation mutants from this series, it could not be recruited to the membrane by FZD5 cotransfection (Fig. 5C). A functional complementation assay with DVL KO HEK293 cells showed that the DVL1(1–438) mutant could, to some extent, rescue TCF/LEF-dependent transcription, as determined by the TopFlash reporter assay (Fig. 5D). Western blotting (WB) analysis revealed that none of the mutants could restore S1490-LRP6 phosphorylation (Fig. 5E). However, the DVL1(1–444) and DVL1(1–461) mutants possibly failed to fold correctly, and their functionality was thereby affected (Fig. 5B and E).

In summary, our data suggest that the N-terminal part of the DEP domain is key for the ability of DVL1 to restore communication between the Wnt3a-receptor complex and downstream machinery. In contrast, numerous other residues controlling DVL1 membrane recruitment and function in the Wnt/PCP pathway are dispensable for this process.

## DISCUSSION

Our study allowed for the first time a detailed analysis of the requirements for individual DVL isoforms and DVL regions for Wnt3a-induced phosphorylation of LRP6, which reflects the formation of signalosomes and the subsequent activation of TCF/LEF-controlled transcription. We took advantage of cell lines with a CRISPR/Cas9-edited genome lacking all three DVL isoforms and focused on the ability of individual DVL variants to restore the response to exogenously added Wnt3a. Confirming the previously published results, we found that DVL is indeed indispensable for the phosphorylation of LRP6 and downstream signaling after activation by the Wnt3a ligand (8, 22).

The rescue experiments also revealed that DVL1 is more potent than DVL2 and DVL3 in the rescue of Wnt3a-induced TopFlash reporter activation and pS1490-LRP6 phosphorylation, used as two complementary readouts of Wnt/β-catenin pathway activation. This suggests that individual DVL isoforms are not fully redundant and may have distinct functions in the Wnt/β-catenin pathway. This observation is in line with the distinct phenotypes of mice with knockouts of the individual DVL isoforms (23, 24). It is not clear why overexpression of DVL3 showed the weakest rescue (borderline rescue of pS1490-LRP6 and no effect on TopFlash reporter activation), but this observation suggests the existence of a DVL-dependent mechanism that connects proximal membrane events (activation of LRP6) and downstream inhibition of the destruction complex. Also, it remains to be elucidated whether a similar observation applies to cell types with different combinations of receptors and coreceptors or whether this feature is intrinsic to DVL3. The latter might be supported by the earlier finding that DVL1 has a much greater ability to induce TCF/LEF-dependent transcription even when it is expressed alone, whereas DVL3 requires coexpression of CK1ε for efficient transactivation (P. Paclíková and O. Bernatík, unpublished data).

One of the most important results of this study is the clear evidence that the DVL1 DEP domain is required for the rescue of cell responsiveness to Wnt3a. It is largely accepted that the DEP domain is indispensable for Wnt/PCP signaling, and multiple point mutations in the DEP domain, required for proper PCP signaling, have been reported. The best-characterized mutations include the *Dsh*^*1*^ mutation in Drosophila (corresponding to K417M in human DVL1 [hDVL1]) (9, 12), the mutation of the tyrosine (Y494 in hDVL1) phosphorylated by Abl kinase (20), and mutations in the DEP-membrane interface (19).

Although the importance of the DVL DEP domain for the PCP pathway is broadly accepted, the role of the DEP domain in Wnt/β-catenin signaling has remained a puzzling issue with a lot of contradictory reports. Most mutations in Drosophila melanogaster Dsh that would influence canonical Wnt signaling are not found in the DEP domain (25). In contrast, all PCP-specific mutations (including *dsh*^*1*^) are located in the DEP domain, impair stable DVL membrane recruitment, and appear to be fully functional in the Wnt/β-catenin pathway (9, 19, 20). In addition, the mutations lacking large portions of the DEP domain can rescue/induce Wnt/β-catenin-dependent phenotypes both in the fly and in Xenopus (9, 10). In mammalian cells, the DEP domain has also been shown to be dispensable for the LRP6 phosphorylation occurring in signalosomes at the membrane (22), and multiple labs, including ours, could clearly show that in wild-type cells, DVL variants lacking the DEP domain were sufficient to induce downstream Wnt/β-catenin signaling (17, 26, 27).

The analysis of DEP domain mutants performed in this study provided evidence that none of the previously described mutants with mutations connected with the deregulation of the PCP pathway, such as K438M (9, 12), membrane binding residue mutations RRRKA (R464A/R465A/R468A/K469A) and H482A/K486A (19, 28), or Y494F (20), is defective in the capacity to rescue the Wnt3a response and activation of Wnt/β-catenin signaling. This is largely in agreement with the situation in Drosophila. In contrast, the deletion of residues at the N terminus of the DEP domain (ΔLPDSG) or mutation of conserved aspartates, required for the interaction with FZD (DIDI mutant), creates a DVL1 mutant completely deficient in the ability to restore the Wnt3a response in DVL KO cells. This suggests that the interaction of the DEP domain with the FZD receptor, reported recently (21), is crucial.

Interestingly, when this paper was being completed, a report by Gammons and colleagues (29) suggested that the DEP domain can dimerize via domain swapping and that this dimerization is crucial for signalosome assembly and Wnt/β-catenin signaling. The DEP domain consists of approximately 90 amino acids and is located in the C-terminal part of the DVL protein. It is composed of three α-helices and four β-sheets (30). The N-terminal α-helix (H1), affected by LPDSG deletion, of the DEP domain is critical for domain swapping, and the same is true for the aspartate (D460K) affected in the DIDI mutant. Our data are in line with this mechanism, both by confirming the importance of the DEP domain and by pinpointing the critical importance of residues involved in domain swapping (29).

**FIG 6:**
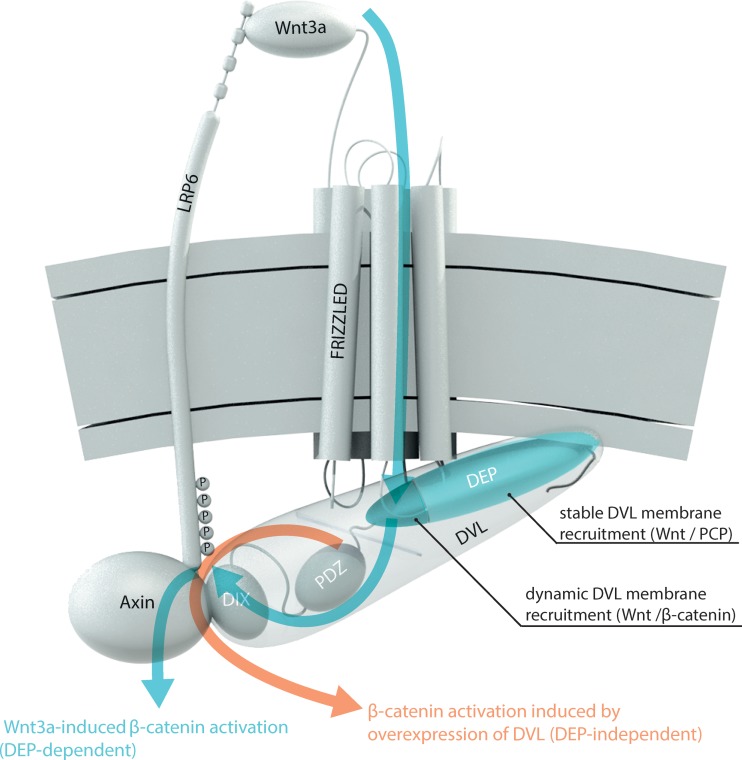
Model of the role of the Dishevelled DEP domain in Wnt/β-catenin signal transduction. Signal transduction is initiated by the interaction of Wnt3a with the extracellular part of LRP6 and Frizzled. DVL is recruited to the membrane and interacts with FZD. The N-terminal part of the DVL DEP domain (dark blue) is necessary for dynamic DVL membrane recruitment to Frizzled after Wnt3a treatment. Other regions of the DEP domain (light blue) that are important for the stable interaction of DVL with FZD and/or the plasma membrane and that are required for the function of DVL in the Wnt/PCP pathway are indispensable in this process. Via its DIX domain, DVL interacts with axin, which brings axin to the membrane complex and leads to LRP6 phosphorylation and downstream Wnt/β-catenin signaling. This Wnt3a-induced β-catenin signal transduction is indicated by blue arrows. Overexpression of DVL, commonly used in the experiments in the Wnt field, can bypass the requirement for the DEP domain in the Wnt/β-catenin pathway by nucleating endogenous DVL and axin (via the DIX domain) and triggering Wnt ligand-independent β-catenin activation. This pathway is indicated by an orange arrow.

We have attempted to summarize the results from this study and from the literature in the model presented in Fig. 6. This model reconciles (i) the older functional data (9, 10) demonstrating that large parts of the DEP domain but not its N terminus are dispensable for its function in Wnt/β-catenin signaling, (ii) the discrepancies between data obtained using endogenous and overexpressed DVL, as well as (iii) differences between the function of the DEP domain in Wnt/PCP and Wnt/β-catenin signaling.

Of note, DVL1(1–502), lacking the C terminus, could rescue the Wnt3a response as well as FL DVL1 could. These data show that the region of DVL1 C terminally from the DEP domain is dispensable for canonical Wnt signaling. These data are in contrast to those from the work of Tauriello et al. (2012), who proposed that the part of DVL1 in the C-terminal direction from the DEP domain binds to FZD intracellular loop 3 and is critical for FZD-DVL1 binding (21). In combination, the results may suggest that the C terminus is not absolutely required but may contribute to the fine-tuning of DVL by its regulatory function. The mechanism of this process will need further experimental work.

In summary, our complementation assays allowed to us clarify some of the long-standing discrepancies in the Wnt field. It largely confirmed the results from the Drosophila model, which suggest that key molecular mechanisms are fully conserved between flies and mammals. It also raises a need to strictly distinguish between the ability to restore Wnt3a-induced events and overexpression-triggered phenotypes, as well as urges the need to distinguish between stable, Frizzled overexpression-induced membrane relocalization of DVL, which is likely more relevant for the PCP pathway, and dynamic DVL-Frizzled interactions, which do manifest differently and cannot be captured by the DVL membrane translocation assay.

## MATERIALS AND METHODS

### Cell culture and transfection.

HEK293 (human embryonic kidney) cells, *DVL1/DVL2/DVL3* KO HEK293 cells (DVL KO HEK293 cells; described elsewhere [13, 14]), HEK293 T-REx cells (WT T-REx cells), and *DVL1/DVL2/DVL3* KO HEK293 T-REx cells (DVL KO T-REx cells) were cultured in Dulbecco's modified Eagle's medium (DMEM; catalog number 41966-029; Gibco, Life Technologies) to which 10% fetal bovine serum (FBS; catalog number 10270-106; Gibco, Life Technologies), 1% penicillin-streptomycin (catalog number XC-A4122/100; Biosera), and 1% l-glutamine (catalog number 25030024; Life Technologies) were added.

For transfection, cells were seeded at 200,000 cells/well in a 24-well plate. On the next day, the cells were transfected using polyethylenimine (PEI) at a concentration of 1 μg/ml and pH 7.4 and a PEI ratio of 6 μl of PEI/1 μg DNA. The mixture of transfected plasmids and PEI was diluted separately in plain DMEM (DMEM without FBS, l-glutamine, and antibiotics) and incubated at room temperature for 15 min, and plasmid DNA and PEI were mixed afterwards. The total transfection mix volume was 50 μl per well. Samples were vortexed, centrifuged, and incubated for 20 min at room temperature before addition to the cells. After 8 h, the medium containing the transfection mix was removed and exchanged for complete DMEM. For WB, cells were treated with Wnt3a for 2 h. For rescue experiments, a concentration of a plasmid carrying DVL of 0.01 μg DNA/well was used, if not stated otherwise. Under each condition, 0.005 μg of a plasmid carrying GFP DNA was cotransfected. The total amount of DNA was equalized to 0.4 μg DNA/well by the use of pcDNA3.1.

In all experiments, cells were treated with 1 μM Porcupine inhibitor LGK974 (catalog number 974-02; Stem RD) to reduce the autocrine secretion of all Wnt ligands. For stimulation of the cells, control or Wnt3a conditioned medium (CM) was used. For the rescue experiments in which analysis was by WB, cells were treated with 500 μl conditioned medium per well for 2 h. For the rescue experiments in which analysis was by the dual-luciferase assay, cells were treated with 200 μl of CM per well for 14 h. In some of the experiments, cells were alternatively stimulated with mouse Wnt3a recombinant protein for 14 h at a concentration of 100 ng/ml (catalog numbers 1324-WN and 645-WN; R&D Systems) and recombinant human R-spondin 1 at 250 ng/ml (catalog number 120-38; PeproTech). Control stimulations were done with 0.1% bovine serum albumin in phosphate-buffered saline (PBS).

### CRISPR/Cas9 generation of DVL1/DVL2/DVL3 KO HEK293 T-REx cells.

Plasmids encoding guide RNAs targeting three isoforms of human DVL genes were used (for hDVL1, ACATGTCGCCGGGCTCGATGCGG, where the TaqI restriction site is underlined; for hDVL2, AAAAAGTACTTGGCGCCCGCGGG; for hDVL3, GCGAGAGCGGCCACGCCGGAGGG). The following plasmids were used: humanized wild-type Cas9 plasmid (plasmid 41815; Addgene) (31) and the PiggyBac system (hygromycin resistance cassette and transposase) (32).

HEK293 T-REx cells (Invitrogen) were cultured according to the manufacturer's instructions and transfected by use of the Lipofectamine 2000 DNA transfection reagent (Thermo Fisher Scientific) with WT human Cas9, PiggyBac Hygro, PiggyBac transposase, and a plasmid carrying guide RNAs targeting human DVL1, DVL2, and DVL3 genes. A stable polyclonal cell line was established by using hygromycin B (catalog number sc-29067; Santa Cruz Biotechnology) at a concentration of 50 μg/ml for 10 days. Isogenic lines were derived using the limiting dilution method. Selection of DVL1 KO was done by PCR. Genomic DNA was isolated by use of the DirectPCR lysis reagent (catalog number 301-C; Viagen Biotech), and then the fragment of genomic DNA was amplified by PCR using DreamTaq DNA polymerase (Thermo Fisher Scientific), forward primer CCTCCTTCAGCAGCATAACC, and reverse primer CGGCATCGTCATTGCTC. Then, the PCR product was cut with TaqI (catalog number ER0671; Thermo Fisher Scientific) and the reaction products were visualized on a 2% agarose gel. The amplified DNA fragment without the Cas9-mediated modification showed two bands (250 nucleotides [nt] and 300 nt) due to the TaqI cut, whereas the PCR product with the TaqI recognition site disrupted was not cut. Cell lines with DVL2 and DVL3 mutants were screened by Western blotting using antibodies to DVL2 (catalog number cs3216; Cell Signaling Technology) and DVL3 (catalog number sc-8027; Santa Cruz Biotechnology). For verification of the targeting, genomic DNA was amplified by PCR, cloned into EcoRV-digested plasmid pcDNA3 (Invitrogen), and sequenced using cytomegalovirus forward primer CGCAAATGGGCGGTAGGCGTG.

### Western blotting, immunocytofluorescence, dual-luciferase assay, and immunoprecipitation.

Cells for the dual-luciferase assay were transfected using PEI (described above), while, in addition to the DVL plasmids, 0.1 μg of the pRLtkLuc plasmid and 0.1 μg of the Super8X TopFlash plasmid per well were cotransfected. The dual-luciferase assay was performed by use of a Dual-Luciferase reporter assay system (catalog number E1960; Promega) according to the manufacturer's instructions. Luminescence was measured by use of a Hidex Bioscan Plate Chameleon luminometer. Data were analyzed with Microsoft Excel and GraphPad Prism software. Immunoblotting and sample preparation were performed as previously described (33). The antibodies used included antibodies to LRP6 (catalog number cs3395), pS1490-LRP6 (catalog number cs2568), DVL2 (catalog number cs3216), and axin1 (catalog number cs2087) from Cell Signaling Technology; actin (C-11; catalog number sc-1615) and DVL3 (catalog number sc-8027) from Santa Cruz Biotechnology; active β-catenin (catalog number 05-665) from Millipore; FLAG M2 (catalog number F1804) from Sigma; and CK1ε (catalog number 610446) from BD Biosciences. For DVL1 antibody testing, antibodies to DVL1 (catalog numbers sc-133525 [Santa Cruz], D3320 and D3570 [Sigma-Aldrich], and cs8025 [Cell Signaling Technology]) were used. Western blots were quantified using ImageJ software. Briefly, the area of peak intensity for pS1490-LRP6 was divided by that for actin, and statistical significance was confirmed by paired Student's *t* test. Quantification was performed when the number of experiments was at least 3.

Immunocytofluorescence was done as previously described (26). Briefly, DVL KO HEK293 cells were transfected with 20 ng of plasmids carrying the corresponding DVL-FLAG and 50 ng of the plasmid carrying V5-FZD5 or hemagglutinin (HA)-axin1, stained with FLAG (catalog number F7425; Sigma), V5 (catalog number R96025; Life Technologies), or HA (catalog number MMS-101R; Covance) antibodies, and visualized with Alexa Fluor 488-conjugated donkey anti-rabbit immunoglobulin (catalog number A21206; Invitrogen) and Alexa Fluor 568-conjugated anti-mouse immunoglobulin (catalog number A-11004; Invitrogen) secondary antibodies. Nuclei were stained with DRAQ5 (catalog number 4084S; Cell Signaling Technology). Images were acquired using a Leica SP8 system. One hundred positive cells per experiment (*n* = 3) were analyzed, and DVL1 membrane recruitment was scored into three categories (strong, weak, and no membrane localization of DVL1).

The immunoprecipitation protocol used was modified from that of Bryja et al. (33). Briefly, DVL KO HEK293 cells were seeded into 10-cm culture plates and transfected with 0.4 μg of plasmid carrying the corresponding DVL1, and the total amount of DNA was equalized to 4 μg by the use of pcDNA3.1. After 24 h of growth, the medium was removed, the cells were washed with PBS, and 1 ml of cold lysis buffer supplemented with protease inhibitors (catalog number 11836145001; Roche Applied Science), phosphatase inhibitors (catalog number 524625; Calbiochem), and 0.1 mM dithiothreitol (catalog number E3876; Sigma) was used per sample. Lysate was collected after 20 min of lysis at 4°C and was cleared by centrifugation at 16.1 × *g* for 20 min. One microgram of antibody was used per sample. Samples with the antibody were incubated for 40 min, and then 20 μl of protein G-Sepharose beads (catalog number 17-0618-05; GE Healthcare) equilibrated in complete lysis buffer was added. Samples were incubated on the carousel overnight and washed 6 times. Forty microliters of 2× Laemmli buffer was added, and the samples were boiled. The antibodies used for immunoprecipitation were axin1 (catalog number cs2087; Cell Signaling Technology) and CK1ε (catalog number 610446; BD Biosciences).

### RT-PCR.

Cells were seeded into a 24-well plate and at 24 h after seeding were starved in 1% FBS–DMEM and treated with the inhibitor LGK974 (1 μM) for 36 h. Wnt3a activation (100 ng/ml; catalog number 1324-WN; R&D Systems) was performed 14 h before the cells were harvested. Total RNA was isolated using an RNeasy minikit (catalog number 74106; Qiagen) according to the manufacturer's instructions. mRNA was transcribed to cDNA (SuperScript II reverse transcriptase; Thermo Fisher Scientific) and analyzed by use of a Sybr green system for *AXIN2* (forward primer, AAAGAGAGGAGGTTCAGATG; reverse primer, GAATAAGTACCAGACCATTGAC; Sigma) using the housekeeping genes *B2M* (forward primer, CACCCCCACTGAAAAAGATG; reverse primer, ATATTAAAAAGCAAGCAAGCAGAA; Sigma) and *ACTIN* (forward primer, TCCCTGGAGAAGAGCTACGA; reverse primer, AGCACTGTGTTGGCGTACAG; Sigma), and the amount of mRNA was calculated as 2^−ΔΔ*CT*^. Statistical significance was confirmed by paired Student's *t* test.

### Plasmids and site-directed mutagenesis.

The plasmids used were described previously and included pcDNA3-Flag-mDVL1; DVL-pcDNA3-Flag-mDVL1 (aa 1 to 502), pcDNA3-Flag-mDVL1 (DVL1 aa 1 to 394), pcDNA3-Flag-mDVL1 (DVL1 aa 1 to 345), pcDNA3-Flag-mDVL1 (DVL1 aa 1 to 250), pcDNA3-Flag-mDVL1 (DVL1 aa 1 to 217) (17) carrying DVL1 with truncations or point mutations; pCDNA3.1-(zeo)-Venus-hDVL1 and pCDNA3.1-Flag-hDVL3 (34); pcDNA3.1-HA-mDVL2 (7); pEGFP-C2 from Clontech; pRLtkLuc from Promega; Super8X TopFlash (35); pEGFP-C1-mDVL2 and pEGFP-C1-hDVL3 (36); pCMV5-3×-FLAG-hDVL2 (37); and pCMV hDvl3-HA (38). Site-directed mutagenesis of pcDNA3-Flag-mDVL1(1–502) was performed by use of a QuikChange II XL site-directed mutagenesis kit (Agilent) according to the manufacturer's instructions. The sequences of all mutants described in this study were verified by sequencing.
